# Phylogenetic microbiota profiling in fecal samples depends on combination of sequencing depth and choice of NGS analysis method

**DOI:** 10.1371/journal.pone.0222171

**Published:** 2019-09-17

**Authors:** Sukithar K. Rajan, Mårten Lindqvist, Robert Jan Brummer, Ida Schoultz, Dirk Repsilber

**Affiliations:** School of Medical Sciences, Örebro University, Örebro, Sweden; Nanjing University, CHINA

## Abstract

The human gut microbiota is well established as an important factor in health and disease. Fecal sample microbiota are often analyzed as a proxy for gut microbiota, and characterized with respect to their composition profiles. Modern approaches employ whole genome shotgun next-generation sequencing as the basis for these analyses. Sequencing depth as well as choice of next-generation sequencing data analysis method constitute two main interacting methodological factors for such an approach. In this study, we used 200 million sequence read pairs from one fecal sample for comparing different taxonomy classification methods, using default and custom-made reference databases, at different sequencing depths. A mock community data set with known composition was used for validating the classification methods. Results suggest that sequencing beyond 60 million read pairs does not seem to improve classification. The phylogeny prediction pattern, when using the default databases and the consensus database, appeared to be similar for all three methods. Moreover, these methods predicted rather different species. We conclude that the choice of sequencing depth and classification method has important implications for taxonomy composition prediction. A multi-method-consensus approach for robust gut microbiota NGS analysis is recommended.

## Introduction

The human gastrointestinal tract (GI) is complex and harbors trillions of microorganisms, mostly consisting of bacteria but also viruses and protozoa. The microbiota plays an important part in gut barrier homeostasis under healthy conditions and a disruption of the gut microbiota composition is known to influence the intestinal physiology negatively [1]. Dysbiosis of the intestinal microbiota has been associated with several diseases, including inflammatory bowel disease [2] and Alzheimer [3]. The composition of these gut microbiota varies along the digestive tract and consists of more than 1000 bacterial species [4]. However, 99% of the gut microbiota is represented by 30–40 bacterial species only [5]. Considering the influence of microbes on their host, an initiative with the goal of identifying and characterizing gut microbiota, The Human Microbiome Project (HMP), was launched in 2008 [6]. A similar project was initiated in Europe, the MetaHIT (2008–2012), with the aim to establish the association between genes of the human gut microbiota and our health and disease, focusing mainly on Inflammatory Bowel Disease (IBD) and obesity [7]. The International Human Microbiome Consortium (IHMC) was a simultaneous project aiming at understanding the role of human gut microbiota in maintaining health and causation of disease, for improvement of treatment and prognosis of diseases [8]. Despite these extended efforts in human-microbiome research, microbial communities are still the dark matter of human health and disease. Since the gut microbiome consists mainly of anaerobic members, the conventional approach of growth media-based culturing is not a feasible option [9]. Finally, complexity and very uneven distribution of species composition, make their investigation challenging.

Culture-independent methods based on targeted sequencing of 5S and 16S ribosomal genes to identify microbes date back three decades [10]. Introduction of next generation sequencing (NGS) to 16S rRNA gene analysis revolutionized microbiome analysis, with a marked improvement in cost and sampling depth [11]. Metagenomics, in the form of direct random shotgun sequencing of environmental DNA, constitutes the current extended application of NGS in microbiome analysis, targeting the complete genomic sequences of all microbiota [12].

Mock bacterial communities have been used in microbiome studies to understand biases introduced during data generation and subsequent data analysis [13]. Previous studies have reported differences in results related to DNA extraction methods, PCR amplification, as well as sequencing platforms. Thus, using a known bacterial community to initially validate analysis pipeline and interpretation of results is a necessary first step [14, 15].

Most metagenomic data analysis pipelines employ a taxonomy classification step in order to understand and estimate microbial abundance and diversity in an environmental sample. In this step, metagenome sequences are screened using relevant reference databases, and the relative abundance of each phylogenetic level is estimated. Most metagenomic classification tools provide multilevel taxonomy indices, some claiming resolution even at strain level [16–18]. Even though the majority of sequences remain unclassified (~65%) [19, 20], the classified sequences cast light on the population structure of respective samples. Scaling the computational repertoire to catch up with the exponential pace in NGS data generation is another overlooking challenge. A major concern is about reaching an optimal sequencing depth in order to extract accurate population-related information, to accurately determine microbiota composition from a sample. Few studies have investigated the consequences of an interaction of methodological choice and applied sequencing depth for conducting metagenomic analyses [18, 21]. Instead, studies comparing methodological approaches have taken different routes of investigation so far. Comparisons regarding use of sequencing platforms and taxonomical classification methods have been reported previously [22, 23]. Jovel et al., compared 16S rRNA gene-based and shotgun-based libraries using OTU tables (for 16S) and lowest common ancestor (LCA) (for shotgun) based taxonomy approaches. Different taxonomy classifiers and reference databases are discussed in their work, but not explicitly compared [22]. Hillmann et al., compared the use of 16S rRNA gene sequencing, shallow shotgun sequencing, and deep whole metagenome sequencing for gut microbiome characterization. They used multiple LCA-based taxonomy classifiers and a common reference database (NCBI RefSeq) [23]. There is a need for a detailed understanding of the influence of different sequencing depths using different classification approaches [24].

The aim of our study is to understand the impact of sequencing depth and reference databases interacting with different choices of classification method on results of phylogenetic classification. We used deep whole metagenome sequencing of human fecal samples to characterize the microbiota composition, to comprehensively compare different taxonomy classification approaches at different sequencing depths, to understand the consequences of choice of sequencing depth and its interaction with choice of method. Our choice of maximum sequencing depth, a full-lane on HiSeq2500 paired-end sequences, generating around 200 million sequence read pairs, relates to the maximum affordable sequencing depth cited in recent studies [19, 20]. The results of this investigation will guide researchers while deciding sufficient depth for a sample to be sequenced, which is cost effective and enables post-sequencing analysis as desired. We used shotgun metagenome sequences from one especially deep sequenced fecal sample, which was subsampled to different sequencing depths. A set of 50 subsamples of the total body of all reads was generated at specific depths and used for taxonomy classification employing three different approaches. Subsequent comparisons of taxonomy of classified sequences at different depths and methods, using default reference databases and a consensus reference database, were implemented using three different classification approaches.

## Materials and methods

### Mock community data and validation of methods

Shotgun genomic sequences from a mixture of 11 bacterial species, spanning 7 genera were sequenced by Jovel et al., using Nextera XT (Illumina) kits [22]. Paired end raw sequencing reads from their study, accessible from Sequence Read Archive portal of NCBI under accession number SRX1075649, were used as mock community data. In our study, this mock community data was subjected to taxonomy classification by three methods, for an initial methods validation, as described below.

### Data generation

A single fecal sample was used as basis for this work. Ethical permit was given by the Regional Ethical Board at Uppsala University (Regionala Etikprövningsnämden Uppsala), 2012/309. Protocols for metagenomic DNA extraction from this sample and sequencing were strictly followed, if not reported otherwise, as reported elsewhere [25]. DNA samples for sequencing were prepared by repeated bead beating and using QIAamp DNA Stool Mini Kit from Qiagen (Art.no: 51504) [26] following the standard protocol, and extracted DNA concentration and quality were measured using Nanodrop. A full lane paired-end DNA sequencing was run using an Ilumina HiSeq 2500 system (Illumina, San Diego, CA, USA). Raw sequences, around 200 million read pairs of 126 bases, are accessible at the Sequence Read Archive (SRA) under accession number PRJNA509325.

### General workflow

**Fig 1:**
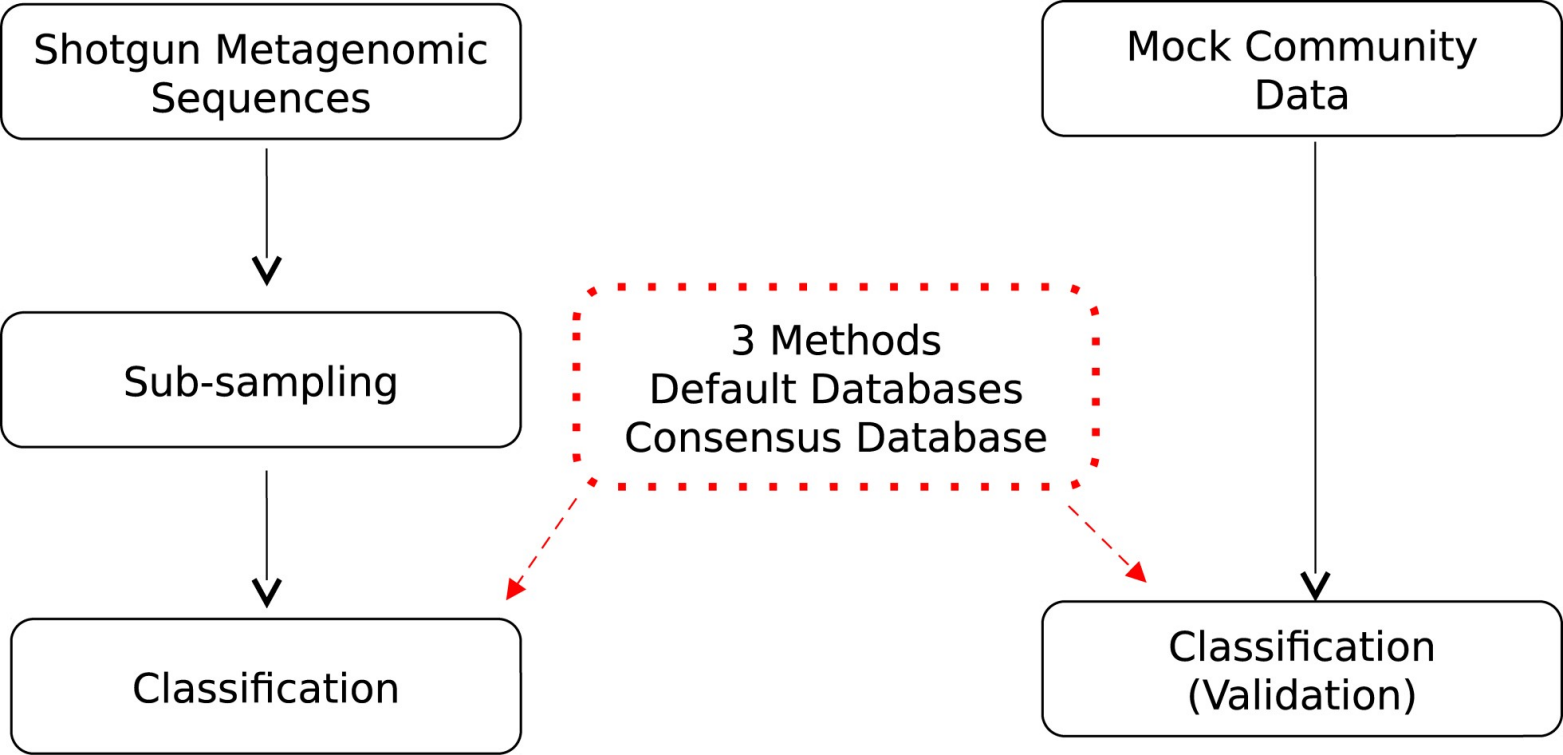
General workflow. Next generation shotgun deep sequences were subsampled to simulate varying sequencing depths. Methods used in this study were validated using mock community data, and subsampled sequences were taxonomically classified using different databases.

Shotgun metagenomic sequence subsampling was conducted to generate simulations of experiments at different sequencing depths. Methods validation was performed using a set of known species genomic sequences. Taxonomic composition was analyzed using three alternative approaches, using default and a consensus reference database. A general overview of the workflow for analyzing subsampled deep sequences is described in Fig 1.

### Simulation of different sequencing depths by subsampling

Metagenomic shotgun DNA sequences were quality checked using FastQC [27], and subsampled to simulate experiments at various sequencing depths before running different taxonomy classifiers. A set of 50 subsamples was generated at specific depths of 5, 10, 20, 40, 80, and 100 million read pairs of the sequenced full lane sample, using the deep sequenced reads (paired-end, around 200 million reads). Subsampling scripts are available at https://git.oru.se/srrn/manuscript_1.

### Sequence classification methods: Similarity based

We used Metaphlan-II, Kraken, and Clark-S taxonomy classifiers in this study [16, 28, 29]. Subsampled shotgun metagenome sequences were used to generate taxonomy classification using these alternative approaches. Standard operating procedures have been followed for each tool, and default settings were used for classifying sequences. Only species with non-zero abundance were reported. As all of these classifiers use a similarity-based approach for read-reference matching, their specific variation in assigning taxonomy is discussed later.

### Data analysis

The subsampled sequences were taxonomically classified using the selected classification approaches separately, at each sequencing depth and using different reference databases, to generate respective species and genus taxonomic identities. 95% confidence intervals of relative abundances of species abundance for all sequencing depths were based on the respective quantiles of their distribution. Species diversity was determined as Shannon diversity, richness (species abundance), and evenness as detailed below. Hereto, the Shannon-Weiner’s index was calculated using the formula [30, 31]:

$$H=-\sum_{i=1}^{n}p_{i}lnp_{i}$$

Where:

H = Shannon-Weiner’s diversity index

n = the number of species

P_i_ = proportion of individuals in species

Species Evenness = H / log (species richness).

We chose principle component analysis–PCA [30] in R (version 3.5.1, Package pcaMethods) as a typical example of high-level analysis to investigate consequences of sequencing depth and methods choice on this level. In order to visualize variation in data, scores plots (PC2 v/s PC1) were assessed. Subsampling, formatting, and taxonomy prediction tasks were automated using in-house scripts written in Bash, R, and/or Python; which are available at https://git.oru.se/srrn/manuscript_1.

### Constructing a consensus database for methods comparison

A consensus database for the classification methods was created based on species which are detectable by all three methods. Genomic sequences of these species were collected and were basis for custom databases. For Kraken and Clark-S, this collection of sequences was used directly. For Metaphlan-II, only clade-genes connected to the species in the consensus collection were used to build a new index with Bowtie2, and the internal database in Metaphlan-II was updated accordingly (https://bitbucket.org/biobakery/metaphlan2/src/default/#markdown-header-customizing-the-database, [16]).

## Results

### Taxonomy prediction using default reference databases

**Fig 2:**
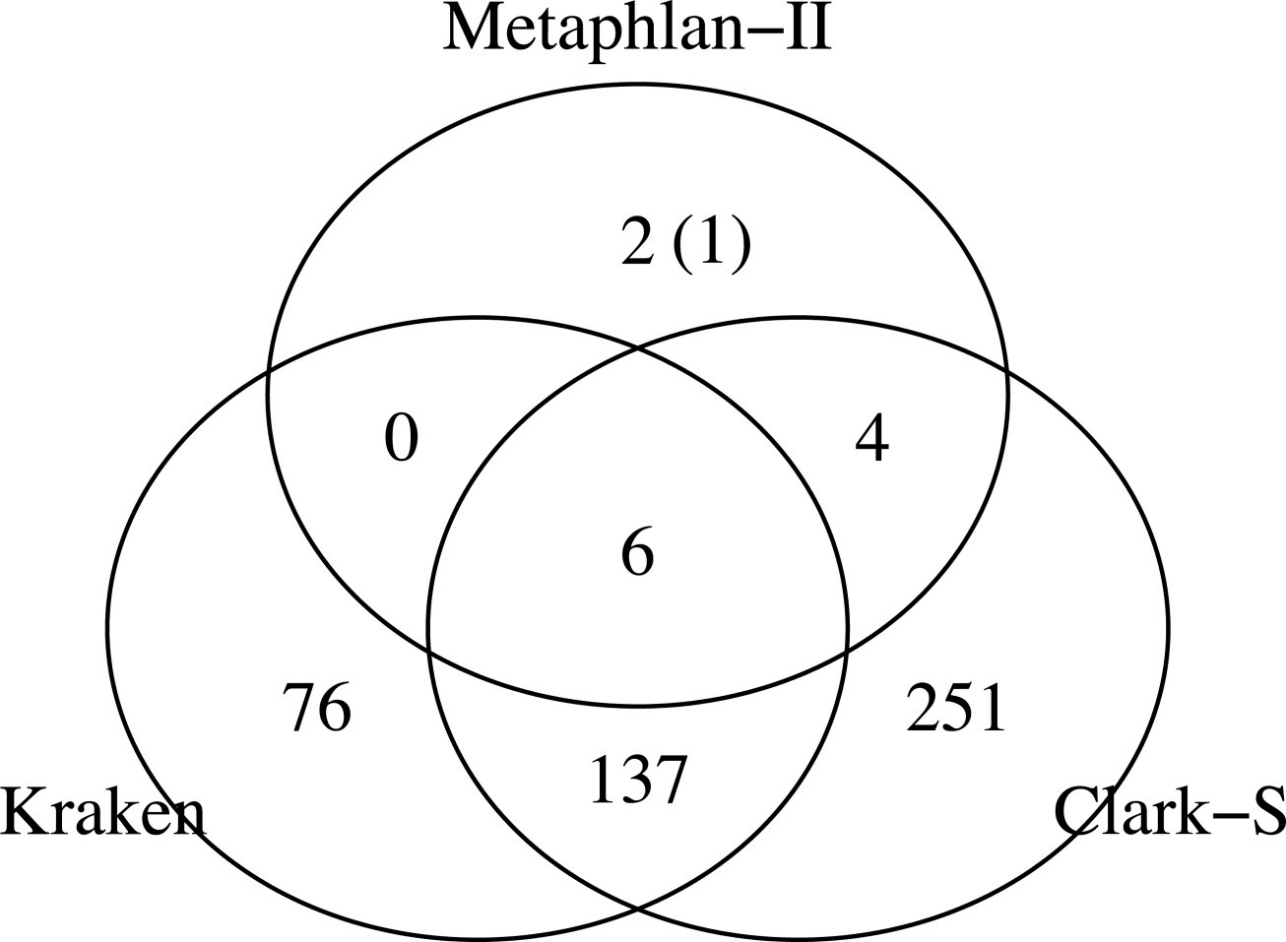
Predicted taxonomical composition of mock community. Abundances of reported species as from the three classification methods. Metaphlan-II classifications were all correct, except one falsely classified. Most of the classifications by Kraken and Clark-S were false positives. The value given in brackets represents an additional species identified.

This study was conducted to examine the influence of the combination of sequencing depth, reference databases, and methods choice on taxonomic classification when analyzing human fecal sample microbiota using shotgun deep sequencing. In order to validate the selected methods, as a first step, a mock community dataset composed of known bacterial species was analyzed using all three taxonomy classification methods using default and a consensus reference database. The mock community population consisted of 11 different species spanning 7 different genera [22]. Taxonomy classifications of this data using the three methods differ considerably as shown in Fig 2. Kraken reported 6 out of 11 species and Clark-S identified 10 out of 11 mock community species, while Metaphlan-II reported all mock community species. Metaphlan-II identified one additional species, while Kraken and Clark predicted many more additional, *i*.*e*. false positive, species. Mock analysis showed precisions of 0.916, 0.027, and 0.025 for Metaphlan-II, Kraken, and Clark-S respectively. The recall rates were 1.0, 0.545, and 0.909 for Metaphlan-II, Kraken, and Clark-S respectively.

Shotgun metagenomic sequences from one especially deep sequenced fecal sample (around 200 million read pairs) were randomly subsampled 50 times for each of the relative sequencing depths of 5, 10, 20, 40, 80, and 100 million read pairs. The sequences of these subsampling datasets were taxonomically classified using different approaches as implemented in the classification softwares Metaphlan-II, Kraken, and Clark-S, at default settings. In these default settings, microbial reference sequences available at NCBI’s RefSeq repository were used by Kraken and Clark-S, while Metaphlan-II uses IMG [32] database, which employs NCBI’s RefSeq as its main source of genomic data, but also additional resources [16, 28, 29]. Genus-specific and species-specific taxonomy information was collated separately from each classification tool. A summary of input and output at each step for each approach is described in Table 1. A detailed description of tools, scripts, and protocols used can be found in the methods section.

**Table 1:** Summary of reads used and classification results for different approaches (full depth).

Method	Metaphlan-II	Clark-S	Kraken
**No. of raw read pairs**	200 M	200 M	200 M
**No. of classified read pairs**	20 M	26 M	40 M
**No. of phylum**	6	33	33
**No. of genus**	55	670	733
**No. of species**	119	1613	1621

M = million

**Fig 3:**
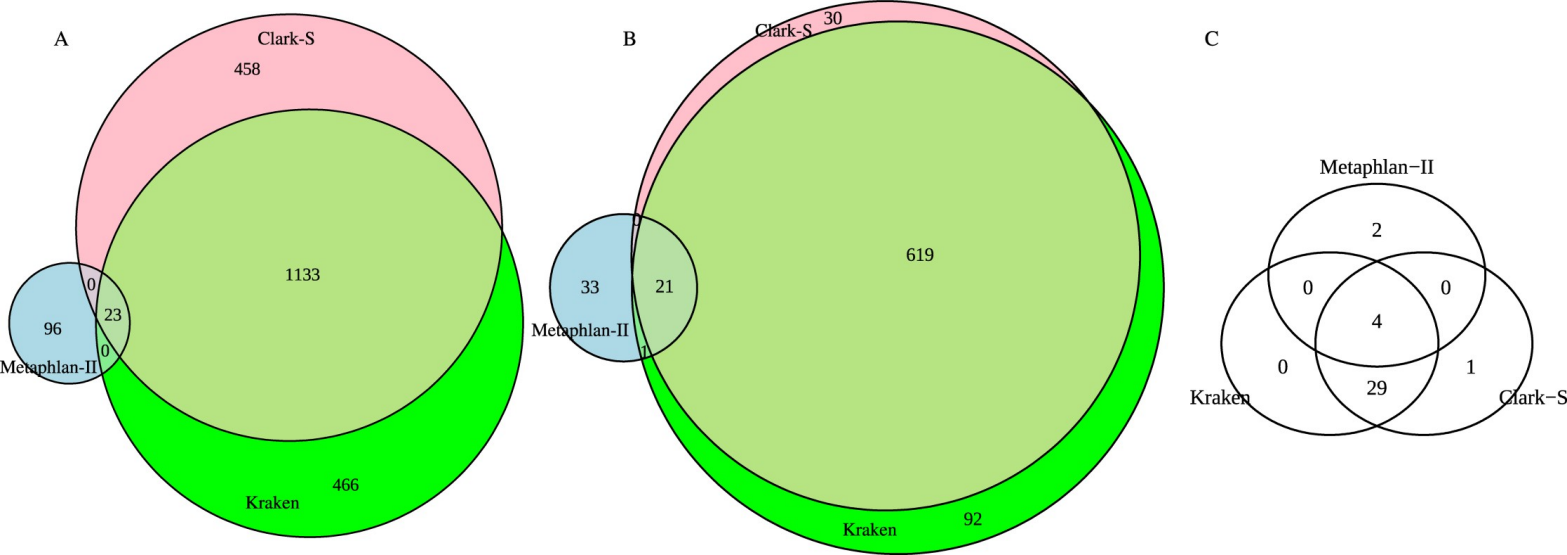
Taxonomical composition predicted for a fecal sample at full depth using 3 approaches. **A**. Abundance of reported species, (**B**) reported genera, and (**C**) reported phyla.

**Fig 4:**
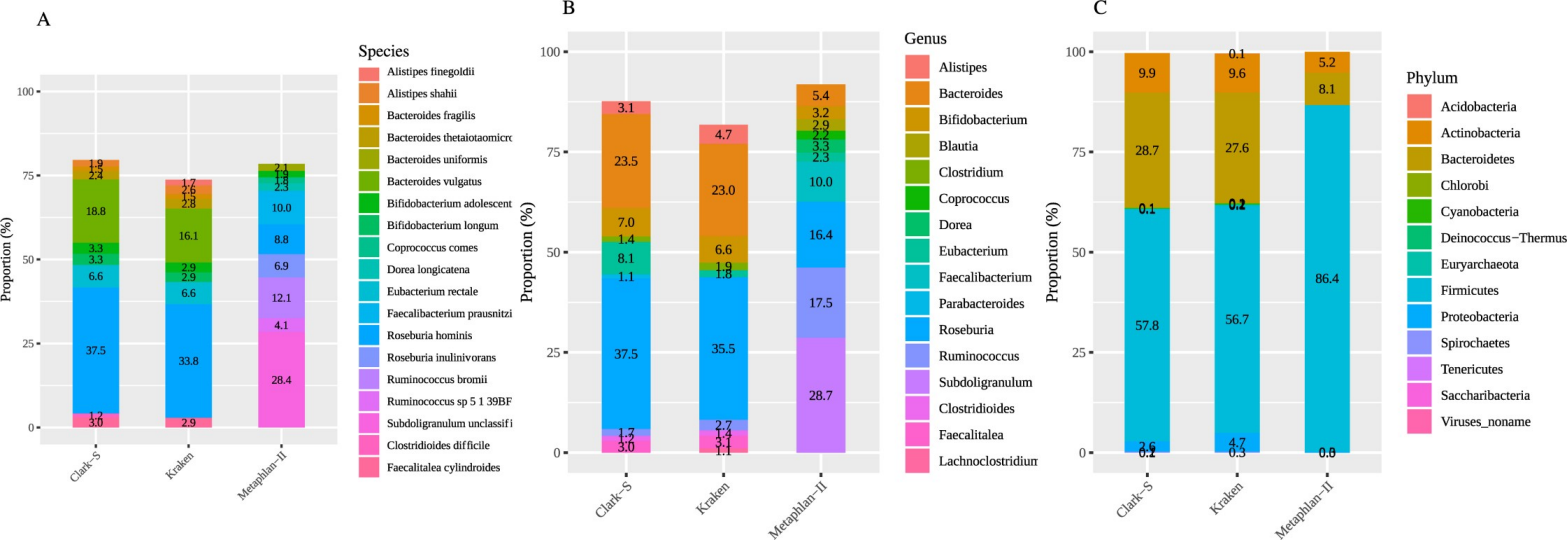
Top-10 taxa predicted at full depth using 3 approaches. **A** Species. **B** Genus. **C** Phyla.

Comparison of taxonomy classifications by the classification tools, based on all 200M read pairs (full-length lane), shows differences in species, genus, and phylum abundance (Figs 3 and 4). Kraken and Clark-S identified 2056 species, which were not identified by Metaphlan-II. Kraken and Clark-S identified 19.33% of Metaphlan-II identified species. Similarly, Kraken and Clark-S recognized 38.18% genera identified by Metaphlan-II. A total of 2175 unique species were identified by all 3 software together; with Metaphlan-II identified species being the lowest, at 5.47%. A collection of 796 unique genera was identified by all three software combined; with Metaphlan-II identified genera being the lowest, at 6.79%. Kraken and Clark-S identified similar phyla, and they did not predict 2 phyla reported by Metaphlan-II. Top species, genera, and phyla assigned by all the approaches showed close similarity in their relative abundances (Fig 4A–4C).

**Fig 5:**
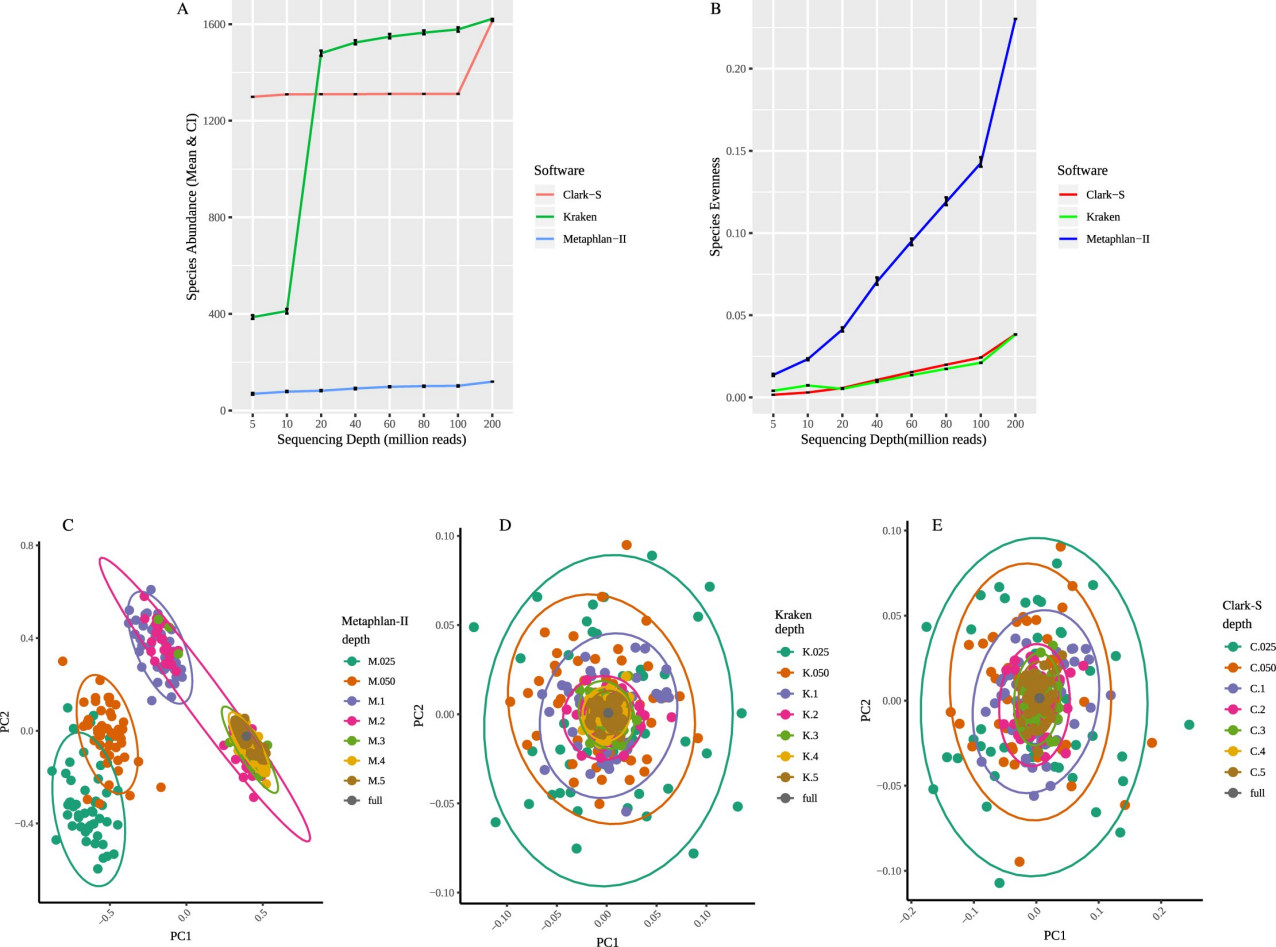
Species diversity prediction as dependent on sequencing depth. **A.** Species richness for different taxonomy classification approaches. Species richness is shown as mean values and 95% confidence intervals as dependent on different sequencing depths. **B**. Species evenness for different taxonomy classification approaches as dependent on different sequencing depths. Principle Component Analysis results for abundances of species identified by **C** Metaphlan-II, **D** Kraken, and **E** Clark-S. First two principal components are shown in scores plots.

The variation in species richness and evenness at different sequencing depths for 50 random subsampling can be found in Fig 5A and 5B. Species abundance across different sequencing depths did not vary a lot for Metaphlan-II and Clark-S, whereas the number of species detected by Kraken correlate to the sequencing depth. Species evenness showed less variation across depths for Clark-S and Kraken, whereas Metaphlan-II showed considerable variation (Fig 5B). Sequencing depth did not seem to affect Metaphlan-II classification generally, even though Metaphlan-II-derived species showed more variation as the species identified were more evenly spread.

Principal component analysis (PCA) is often used in microbiota analysis as it allows to compress the information about the most varying species or genera into few principal components for further analysis. Applied to our classification results, PCA score plots of species abundances at different depths for three classifiers showed similar patterns for Kraken and Clark-S, meanwhile Metaphlan-II showed large species variation for different depths. PCA scores plots for Metaphlan-II show species variation at higher sequencing depths centering around the full-lane, while the scores are clustered differently for lower sequencing depths. Kraken and Clark-S showed a wider spread in species grouping (Fig 5C–5E) in contrast to Metaphlan-II.

In summary, Metaphlan-II-derived classifications constituted 5.47% of total number of species predicted by all classification methods. Around 19% of species predicted by Metaphlan-II overlapped with species identified by the other classification methods. Similarly, 6.79% of total genera predicted were accounted for by Metaphlan-II, and 38.18% of these genera were predicted by the other methods. Both Kraken and Clark-S reported similar phyla, whereas, Metaphlan-II reported different phyla in lower abundance. Prominent phyla reported by all three approaches remained same, with varying abundance; where Kraken and Clark-S showed a closer pattern when it comes to genus and species.

### Taxonomy prediction using the consensus database

**Fig 6:**
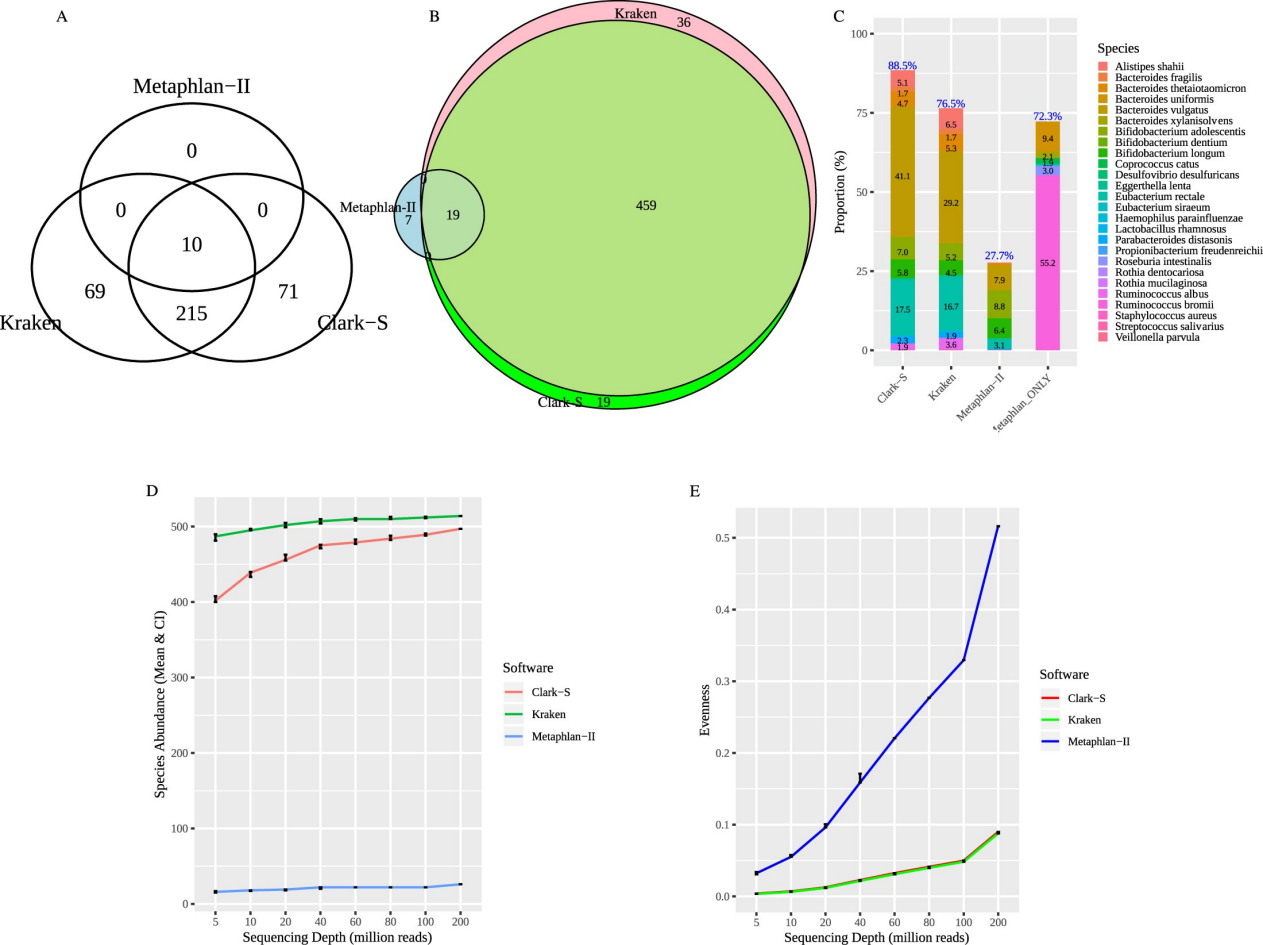
Taxonomical analysis using the consensus database. **A** Representation of predicted species abundance using Metaphlan-II, Clark-S, and Kraken, based on mock community data. **B** Predicted species abundance representation using full depth of fecal sample (200 million read pairs). **C** Composition of the 19 predicted species common to all methods, and specific for Metaphlan-II. The bar named Metaphlan_ONLY represents the species predicted by Metaphlan-II alone, whereas the other three bars represent the common species predicted by each method and their respective proportion. Species with abundance proportion below 1% are not shown in the figure. The sum of abundance proportion for each method is shown at the top of each bar. **D** Species richness for different taxonomy classification approaches at different sequencing depths. Species richness is shown as mean values and 95% confidence intervals (shown as error bars). **E** Species evenness for different taxonomy classification approaches as dependent on different sequencing depths (error bars depict 95% confidence intervals).

Taxonomy classification using a consensus database was conducted to investigate the effect of different databases in taxonomy classification. In order to validate the methods, we analyzed again the mock community of known species [22] using all three methods. The taxonomy classification of this data using the consensus database showed significant differences among the methods. All the methods identified 10 out of 11 mock community species, while Kraken and Clark-S identified many false positive species as shown in Fig 6A. Taxonomy classification of full-lane sequences was performed by all three methods using the consensus database, and their abundance and overlap can be obtained from Fig 6B. For the 19 species commonly predicted by all three methods, these turned out to be the most abundant for Kraken and Clark-S. For Metaphlan-II, these common predicted species only amount to 27.7%. Instead, the 7 species predicted by Metaphlan-II only, represent 72.3% (Fig 6C). Hence, for Metaphlan-II, the 7 species which were not predicted by other methods were the most abundant ones. Among these, *Ruminococcus bromii* (55.2%) and *Bacteroides uniformis* (9.4%) were the most abundant species which were detected by Metaphlan-II alone (Fig 6B and 6C). Subsampled shotgun metagenomic sequences at different sequencing depths were subjected to taxonomy classification using the consensus reference database by all three methods. Species abundance across different sequencing depths did not vary much for Metaphlan-II, whereas, there was a gradual increase in number of predicted species for Kraken and Clark-S, as observed before for using the default reference databases (Fig 5A and Fig 6D). Also, patterns of evenness in predicted species composition as dependent on sequencing depth look similar (Fig 5B and Fig 6E). Predicted species abundance by each method shows similar pattern compared to using default reference databases. The number of species predicted for subsampled shotgun sequences using the consensus reference database was reduced to about 20% when compared to the results using the default reference database (Fig 6B and 6D). A summary of input and output at each step for each approach using consensus database is described in Table 2.

**Table 2:** Summary of read pairs used and prediction results using consensus database.

Method	Metaphlan-II	Clark-S	Kraken
**No. of raw read pairs**	200 M	200 M	200 M
**No. of classified read pairs**	5 M	6 M	8 M
**No. of phylum**	4	19	18
**No. of genus**	19	188	198
**No. of species**	26	498	514

All classifiers performed differently in terms of their computational power, processing speed, and classification results. A performance comparison of three methods is given in Table 3.

**Table 3:** Performance comparison of different methods.

Variables	Metaphlan -II	Clark-S	Kraken
**Time (T)**	2xT	1xT	1xT
**No of species (S)**	0.1xS	1.2xS	1xS
**RAM/processors (P)**	2xP	1xP	1xP

## Discussion

This study investigates the significance of varying sequencing depth in microbiome NGS analysis by comparing taxonomy classification across three standard phylogenetic classification methods at different depths and using different reference databases. We selected taxonomy classifiers, which are openly accessible, stand-alone operative, and capable of delivering species-level phylogenetic classification resolution. A publicly available mock community sequence dataset was used for initial validation of the classification methods. Shotgun metagenomic sequences from a fecal sample were subsampled and used to characterize the microbiome at varying sequencing depths using different reference databases. This study demonstrates large and relevant differences in taxonomy classification based on method choices, reference databases, and sequencing depth.

Analysis of a sample with known origin (mock community) revealed large differences in taxonomy classification between the three methods chosen. At species-level resolution, Metaphlan-II identified all species in the mock community along with an extra species. The extra species also belongs to one of the mock community’s genera. Clark-S identified almost all mock species, together with many more other species (Fig 2). Kraken identified around half of mock community species along with many more other species (Fig 2). The different methods’ performances regarding species identification using mock community data seem similar to the observed differences for classifying microbiome sequences as further discussed below.

Approximately half of species identified by Kraken and Clark-S in addition to those found by Metaphlan-II were found to be shared between the former. Part of the explanation to these findings likely lies in the difference in taxonomy assignment strategy used by Metaphlan-II versus Kraken and Clark-S. Metaphlan-II uses a preselected clade-specific marker gene database, compiled from IMG database, for taxonomy assignment containing approximately 1300 predictable species [16]. Kraken and Clark-S base their classification directly on NCBI Reference Sequence Database with around 8000 species. Only half of all additional species identified by Kraken and Clark-S were in principle predictable by Metaphlan-II. In addition, as both Clark-S and Kraken use a *k-mer* based database for taxonomy assignment, the risk of identifying species that are in proximity to the mock community taxonomy seems enlarged and needs further investigation.

In the main part of this study, sequencing of a fecal sample shotgun NGS analyses served as a realistic example to demonstrate the impact of methods choice and sequencing depth. Second, our choice of sequencing depth, a full-lane on HiSeq2500 of paired-end sequencing, exaggerates the normal affordable sequencing depth in recent studies. At that maximum sequencing depth, most accessed parameters in our study reached approximate plateau, one exception being species evenness. More realistic sequencing depths are assessed from subsampling of the original full lane dataset. Comparison of taxonomical assignments showed considerable variation in genera and species reported when using all data, i.e. all sequence read pairs, across all three methods. While Kraken and Clark-S shared identical phyla, of these only the most prominent two were identified by Metaphlan-II. All three methods identified largely different proportions of reported phyla (for exact proportions, refer to Fig 4C). Kraken and Clark-S displayed significant shares of both genus and species-specific classification, while Metaphlan-II presented only a few representatives in both lists (Figs 3, 4, 5A and 5B). The excessive number of species reported by Clark-S and Kraken compared to Metaphlan-II mirrors the analysis of the mock community (Fig 2). Kraken and Clark-S detected identical phyla, but varied around 10% at genus level, and around 30% at species level. Metaphlan-II showed less than 10% similarity at each taxon level in comparison to Kraken and Clark-S. With increasing sequencing depth, Kraken and Clark-S converge at species levels but diverge from Metaphlan-II. Both Kraken and Clark-S report many more genus and species than Metaphlan-II. A possible explanation to this might lie in the *k-mer* database approach of both methods. Query sequences are fractionized to *k-mers* of 31 bp length for both methods, which is the maximum default length. The frequency of *k-mers* in a genomic sequence or region can be used as a signature of the underlying sequences. Comparison of these frequency signatures are widely used in alignment-free sequence analysis methods [30].

Higher level statistical analysis results in microbiome analysis are also influenced by methods choice and sequencing depth. For example, bacterial diversity analysis is widely used to better understand the population as a whole and its specific characteristics using measures like species evenness and richness. Among the three methods compared, Metaphlan-II showed the lowest richness and highest evenness for reported species (Fig 5A and 5B). Our study demonstrates varying degrees of species diversity predicted for different sequencing depths. Principal component analysis is another frequently used approach to compress information about prominent varying elements in a dataset. Principal components of species abundances reported by Metaphlan-II differed largely in their diversity pattern in comparison to Kraken and Clark-S species. Fig 5C–5E show the spread of prominent varying elements, representing main components of species compositional patterns at different sequencing depths for all three methods. Fig 5C shows a clear pattern of variance among prominent components. Across different sequencing depths, prominent species components at higher sequencing depth appear to be converge, whereas these components appear to be diverging at lower sequencing depths.

The default implementation of the three selected methods uses different reference databases. Metaphlan-II uses IMG database which employs NCBI’s RefSeq sequences as its main source of genomic data with additional information from several other databases potentially missing from NCBI’s RefSeq [32]. Kraken and Clark-S, on the other hand, relay entirely on NCBI’s RefSeq sequences. In order to compare species detectable by all three methods, we created a reference database of consensus species. Thus, 559 unique species spanning all three methods were selected for consensus database construction. Using this approach, all three methods predicted the same species using the mock community data, while Clark-S and Kraken predicted many false positives (Fig 6A). In the fecal sample data, (see Table 2), the taxonomy classification using full-lane sequences predicted 19 common species with varying abundance. Moreover, Metaphlan-II predicted 7 species not reported by Kraken and Clark-S, which were even considered most abundant (Fig 6C). This difference in results can now solely be attributed to different classification approaches in our methods. Taxonomy prediction results for the subsampled shotgun sequences as a factor of sequencing depth were reduced to 20% in abundance using the consensus reference database (Fig 6D and 6E). Thus, the taxonomy predictions for mock community data and subsampled shotgun sequences, using a consensus database by all three methods showed a similar prediction pattern compared to using default reference databases.

In the following, we speculate about why the different approaches for phylogenetic classification used in the three methods under investigation might lead to the observed large variation of species classification. In principle, sequence classification and taxonomy assignment methods depend heavily on identifying similarity between a query sequence and a collection of annotated sequences in databases of bacterial species complete genomes. “Clustering-first” and “assignment-first” are two possible classification strategies used by common classifiers [20] and also for our chosen methods. In a clustering-first approach, initially, sequences are grouped based on their similarity and taxonomically classified using reference databases at a later step. Metaphlan-II, QIIME [33], and Mothur [34] are pipelines that use this approach, which results in identifying a comparatively small number of phylogenetic taxa. Specifically, Metaphlan-II estimates the relative abundance of microbes by mapping query sequences against a reduced set of clade-specific marker genes, which are preselected coding sequences, identifying clades at species level or higher [16]. In Metaphlan-II, the full consensus sequences of the clustered reads are aligned to reference database using Bowtie-2 aligner [35], which requires much time and computational power. In contrast, Kraken and Clark-S construct a *k-mer* based reference database prior to taxonomy assignment. *K-mers* are then mapped against reference sequence databases. The power consuming part in both Kraken and Clark-S is mainly attributed to one-time creation or loading of the reference databases and compilation of *k-mer* database. While Kraken uses the lowest common ancestor (LCA) based approach, Clark-S uses a spaced *k-mer* based approach. Both Kraken and Clark-S require high random-access memory (RAM), for the *k-mer* database. The *k-mer* length is likely a main meta-parameter that could influence the specificity of reported genera and species, and future studies are needed to investigate this [36]. More specifically, longer *k-mer* lengths could deliver more specific taxonomy classifications.

Concluding, the variation in the number of species reported by different methods could be attributed both to the differences in taxonomy assignment strategy and reference databases used by the methods. Whereas total number of predicted species change mostly for using different reference databases, patterns of predictions compared among the three methods remain similar. This is with regard to both overall species richness and evenness as well as regarding results for the mock community.

Several motivations led us in our choice of methods: First, the three approaches were chosen due to their ability to detect taxonomical units at species-level resolution. In addition, our choice represents different classification approaches (gene-set based, LCA based, and spaced *k-mer* based). Many other tools, for example, Mothur [34], QIIME [33], MG-RAST [37], and Kaiju [38] mostly deliver genus-level resolution and partly species-level. Jovel et al., compared sequencing depth of shotgun reads using LCA methods and NCBI RefSeq [22], while Hillmann et al., compared 16S rRNA reads and shallow shotgun reads using LCA methods and NCBI RefSeq [23]. Our study elaborates the impact of sequencing depth by rigorously comparing taxonomical classification approaches at varying sequencing depth using different reference databases.

All tools used in this study are publicly available and follow open source policy, i.e., they are reported and documented sufficiently for anyone to reproduce a similar analysis. We chose to compare three common methods with fixed, default parameters using default and a consensus reference sequence database. A much shallower or deeper sequencing depth, or a change in choice of method settings might have presented a different result. Using other reference sequence databases like Microbial Genome Database for Comparative Analysis (MBGD) [39] or Bacterial Isolate Genome Sequence Database (BIGSdb) [40] or a combination of all these would have possibly led to different phylogenetic profiles. A taxonomic profile based on a more comprehensive gene catalogue [41] might have presented a more accurate prediction. It might be worth trying to do similar analyses through a larger collection of additional taxonomy assignment tools, for example Kaiju [38] or MetAmos [42], and compare their results and performance with this study.

Availability of alternate public mock community datasets will facilitate method optimization attempts, apart from teaching and training. However, none as large and complex as microbiome of fecal sample are in public domain [14, 22, 43]. A more realistically distributed mock community data set would be desirable to validate classification methods. This would enable a better understanding about the prediction potential, particularly with regards to false positives and false negatives among competing methods.

In the light of our findings and with growing computational infrastructure, we propose to consider employing competing taxonomy classification prediction methods, compare and combine their findings to reach a robust consensus result.

It is known that, in addition to sequencing depth and choice of analysis methods, main contributions to variability in results can be attributed to both sampling techniques and DNA extraction methods, as well as details of library preparation. Impact of these additional factors has been studied elsewhere [44, 45]. For our experimental data, a bead-beating Qiagen kit approach was employed, as discussed being optimal in these works.

Regarding further developments of high-level analysis methods, building microbiota protein interaction networks and predicting microbiota metabolic pathways will take us further ahead in understanding the core of bacterial influence in maintaining gut health [46–48]. Also, these will depend on sequencing depth, reference databases, and methodological choices. However, the topological and or functional characteristics of such networks or the enrichment signals of such pathways and or gene sets might prove more stable with respect to the choice of classification tools, and so at reasonable intermediate sequencing depth.

In our study, we investigated the impact of methods choice, reference databases, and sequencing depth and their interaction with respect to taxonomic composition of fecal microbiome. Species abundance reported, using 60 to 80 million sequencing read pairs, show close proximity to the species abundance reported using full-lane sequences. We conclude proposing a multiple-methods approach for robust microbiota analysis results.

## Abbreviations

Bp: base pairs
NGS: Next Generation Sequencing
DNA: Deoxy ribonucleic acid
OTU: Operational Taxonomic Unit
LCA: Lowest Common Ancestor
KEGG: Kyoto Encyclopedia of Genes and Genomes
KO: KEGG Orthology
COG: Cluster of Orthologous Groups
BIOM: Biological Observation Matrix
PCA: Principal Component Analysis
NCBI: National Center for Biotechnology Information
RAM: Random access memory
MBGD: Microbial Genome Database for Comparative Analysis
BIGSdb: Bacterial Isolate Genome Sequence Database

## References

[1] Bull MJ, Plummer NT. Part 1: The Human Gut Microbiome in Health and Disease. Integr Med (Encinitas). 2014;13(6):17–22. Epub 2016/01/16. 26770121 PMC4566439

[2] Natividad JM, Verdu EF. Modulation of intestinal barrier by intestinal microbiota: pathological and therapeutic implications. Pharmacol Res. 2013;69(1):42–51. Epub 2012/10/24. 10.1016/j.phrs.2012.10.007 . 23089410

[3] Halfvarson J, Brislawn CJ, Lamendella R, Vazquez-Baeza Y, Walters WA, Bramer LM, et al Dynamics of the human gut microbiome in inflammatory bowel disease. Nat Microbiol. 2017;2:17004 Epub 2017/02/14. 10.1038/nmicrobiol.2017.4 28191884 PMC5319707

[4] Larroya-Garcia A, Navas-Carrillo D, Orenes-Pinero E. Impact of gut microbiota on neurological diseases: Diet composition and novel treatments. Crit Rev Food Sci Nutr. 2018:1–15. Epub 2018/06/06. 10.1080/10408398.2018.1484340 . 29870270

[5] Savage DC. Microbial ecology of the gastrointestinal tract. Annu Rev Microbiol. 1977;31:107–33. Epub 1977/01/01. 10.1146/annurev.mi.31.100177.000543 . 334036

[6] Icaza-Chavez ME. [Gut microbiota in health and disease]. Rev Gastroenterol Mex. 2013;78(4):240–8. Epub 2013/12/03. 10.1016/j.rgmx.2013.04.004 . 24290319

[7] Rup L. The human microbiome project. Indian J Microbiol. 2012;52(3):315 Epub 2013/09/03. 10.1007/s12088-012-0304-9 23997318 PMC3460114

[8] Blanco-Miguez A, Gutierrez-Jacome A, Fdez-Riverola F, Lourenco A, Sanchez B. MAHMI database: a comprehensive MetaHit-based resource for the study of the mechanism of action of the human microbiota. Database (Oxford). 2017;2017 Epub 2017/01/13. 10.1093/database/baw157 28077565 PMC5225402

[9] Lewis CM Jr., Obregon-Tito A, Tito RY, Foster MW, Spicer PG. The Human Microbiome Project: lessons from human genomics. Trends Microbiol. 2012;20(1):1–4. Epub 2011/11/25. 10.1016/j.tim.2011.10.004 22112388 PMC3709440

[10] Weinstock GM. Genomic approaches to studying the human microbiota. Nature. 2012;489(7415):250–6. Epub 2012/09/14. 10.1038/nature11553 22972298 PMC3665339

[11] Olsen GJ, Lane DJ, Giovannoni SJ, Pace NR, Stahl DA. Microbial ecology and evolution: a ribosomal RNA approach. Annu Rev Microbiol. 1986;40:337–65. Epub 1986/01/01. 10.1146/annurev.mi.40.100186.002005 . 2430518

[12] Oulas A, Pavloudi C, Polymenakou P, Pavlopoulos GA, Papanikolaou N, Kotoulas G, et al Metagenomics: tools and insights for analyzing next-generation sequencing data derived from biodiversity studies. Bioinform Biol Insights. 2015;9:75–88. Epub 2015/05/20. 10.4137/BBI.S12462 25983555 PMC4426941

[13] Thomas T, Gilbert J, Meyer F. Metagenomics—a guide from sampling to data analysis. Microb Inform Exp. 2012;2(1):3 Epub 2012/05/17. 10.1186/2042-5783-2-3 22587947 PMC3351745

[14] Yeh YC, Needham DM, Sieradzki ET, Fuhrman JA. Taxon Disappearance from Microbiome Analysis Reinforces the Value of Mock Communities as a Standard in Every Sequencing Run. mSystems. 2018;3(3). Epub 2018/04/10. 10.1128/mSystems.00023-18 29629423 PMC5883066

[15] Brooks JP, Edwards DJ, Harwich MD, Rivera MC, Fettweis JM, Serrano MG, et al The truth about metagenomics: quantifying and counteracting bias in 16S rRNA studies. Bmc Microbiology. 2015;15 ARTN 66 10.1186/s12866-015-0351-6 PubMed PMID: WOS:000353191800001. 25880246 PMC4433096

[16] Segata N, Waldron L, Ballarini A, Narasimhan V, Jousson O, Huttenhower C. Metagenomic microbial community profiling using unique clade-specific marker genes. Nat Methods. 2012;9(8):811–4. Epub 2012/06/13. 10.1038/nmeth.2066 22688413 PMC3443552

[17] Smith DP, Peay KG. Sequence depth, not PCR replication, improves ecological inference from next generation DNA sequencing. PLoS One. 2014;9(2):e90234 Epub 2014/03/04. 10.1371/journal.pone.0090234 24587293 PMC3938664

[18] Walsh AM, Crispie F, O'Sullivan O, Finnegan L, Claesson MJ, Cotter PD. Species classifier choice is a key consideration when analysing low-complexity food microbiome data. Microbiome. 2018;6 ARTN 50 10.1186/s40168-018-0437-0 PubMed PMID: WOS:000428216500003. 29554948 PMC5859664

[19] Siegwald L, Touzet H, Lemoine Y, Hot D, Audebert C, Caboche S. Assessment of Common and Emerging Bioinformatics Pipelines for Targeted Metagenomics. Plos One. 2017;12(1). ARTN e0169563 10.1371/journal.pone.0169563 PubMed PMID: WOS:000391621500068. 28052134 PMC5215245

[20] Bazinet AL, Cummings MP. A comparative evaluation of sequence classification programs. Bmc Bioinformatics. 2012;13 Artn 92 10.1186/1471-2105-13-92 PubMed PMID: WOS:000308069400001. 22574964 PMC3428669

[21] Fumagalli M. Assessing the effect of sequencing depth and sample size in population genetics inferences. PLoS One. 2013;8(11):e79667 Epub 2013/11/22. 10.1371/journal.pone.0079667 24260275 PMC3832539

[22] Jovel J, Patterson J, Wang W, Hotte N, O'Keefe S, Mitchel T, et al Characterization of the Gut Microbiome Using 16S or Shotgun Metagenomics. Frontiers in Microbiology. 2016;7 ARTN 459 10.3389/fmicb.2016.00459 PubMed PMID: WOS:000374371700001. 27148170 PMC4837688

[23] Hillmann B, Al-Ghalith GA, Shields-Cutler RR, Zhu Q, Gohl DM, Beckman KB, et al Evaluating the Information Content of Shallow Shotgun Metagenomics. mSystems. 2018;3(6). Epub 2018/11/18. 10.1128/mSystems.00069-18 30443602 PMC6234283

[24] Allali I, Arnold JW, Roach J, Cadenas MB, Butz N, Hassan HM, et al A comparison of sequencing platforms and bioinformatics pipelines for compositional analysis of the gut microbiome. BMC Microbiol. 2017;17(1):194 Epub 2017/09/15. 10.1186/s12866-017-1101-8 28903732 PMC5598039

[25] Michel PO, Degen C, Hubert M, Baldi L, Hacker DL, Wurm FM. A NanoDrop-based method for rapid determination of viability decline in suspension cultures of animal cells. Anal Biochem. 2012;430(2):138–40. Epub 2012/09/11. 10.1016/j.ab.2012.08.028 . 22960013

[26] Vandenberg N, van Oorschot RA. Extraction of human nuclear DNA from feces samples using the QIAamp DNA Stool Mini Kit. J Forensic Sci. 2002;47(5):993–5. Epub 2002/10/02. . 12353586

[27] S. A. FastQC: a quality control tool for high throughput sequence data 2010 [cited 2018 08.29]. Available from: https://www.bioinformatics.babraham.ac.uk/projects/fastqc/.

[28] Ounit R, Wanamaker S, Close TJ, Lonardi S. CLARK: fast and accurate classification of metagenomic and genomic sequences using discriminative k-mers. BMC Genomics. 2015;16:236 Epub 2015/04/17. 10.1186/s12864-015-1419-2 25879410 PMC4428112

[29] Wood DE, Salzberg SL. Kraken: ultrafast metagenomic sequence classification using exact alignments. Genome Biol. 2014;15(3):R46 Epub 2014/03/04. 10.1186/gb-2014-15-3-r46 24580807 PMC4053813

[30] Anderson MJ. A new method for non-parametric multivariate analysis of variance. Austral Ecol. 2001;26(1):32–46. 10.1046/j.1442-9993.2001.01070.x PubMed PMID: WOS:000167002000004.

[31] Nudds TD. Variation in Richness, Evenness, and Diversity in Diving and Dabbling Duck Guilds in Prairie Pothole Habitats. Can J Zool. 1983;61(7):1547–50. 10.1139/z83-208 PubMed PMID: WOS:A1983RD15800016.

[32] Markowitz VM, Chen IM, Palaniappan K, Chu K, Szeto E, Grechkin Y, et al IMG: the Integrated Microbial Genomes database and comparative analysis system. Nucleic Acids Res. 2012;40(Database issue):D115–22. Epub 2011/12/24. 10.1093/nar/gkr1044 22194640 PMC3245086

[33] Caporaso JG, Kuczynski J, Stombaugh J, Bittinger K, Bushman FD, Costello EK, et al QIIME allows analysis of high-throughput community sequencing data. Nature Methods. 2010;7(5):335–6. 10.1038/nmeth.f.303 PubMed PMID: WOS:000277175100003. 20383131 PMC3156573

[34] Schloss PD, Westcott SL, Ryabin T, Hall JR, Hartmann M, Hollister EB, et al Introducing mothur: Open-Source, Platform-Independent, Community-Supported Software for Describing and Comparing Microbial Communities. Appl Environ Microb. 2009;75(23):7537–41. 10.1128/Aem.01541-09 PubMed PMID: WOS:000271944800028. 19801464 PMC2786419

[35] Langmead B. Aligning short sequencing reads with Bowtie. Curr Protoc Bioinformatics. 2010;Chapter 11:Unit 11 7. Epub 2010/12/15. 10.1002/0471250953.bi1107s32 21154709 PMC3010897

[36] Koslicki D, Falush D. MetaPalette: a k-mer Painting Approach for Metagenomic Taxonomic Profiling and Quantification of Novel Strain Variation. mSystems. 2016;1(3). Epub 2016/11/09. 10.1128/mSystems.00020-16 27822531 PMC5069763

[37] Keegan KP, Glass EM, Meyer F. MG-RAST, a Metagenomics Service for Analysis of Microbial Community Structure and Function. Methods Mol Biol. 2016;1399:207–33. Epub 2016/01/23. 10.1007/978-1-4939-3369-3_13 . 26791506

[38] Menzel P, Ng KL, Krogh A. Fast and sensitive taxonomic classification for metagenomics with Kaiju. Nat Commun. 2016;7:11257 Epub 2016/04/14. 10.1038/ncomms11257 27071849 PMC4833860

[39] Uchiyama I, Mihara M, Nishide H, Chiba H. MBGD update 2015: microbial genome database for flexible ortholog analysis utilizing a diverse set of genomic data. Nucleic Acids Res. 2015;43(Database issue):D270–6. Epub 2014/11/16. 10.1093/nar/gku1152 25398900 PMC4383954

[40] Jolley KA, Bray JE, Maiden MCJ. Open-access bacterial population genomics: BIGSdb software, the PubMLST.org website and their applications. Wellcome Open Res. 2018;3:124 Epub 2018/10/23. 10.12688/wellcomeopenres.14826.1 30345391 PMC6192448

[41] Li J, Jia H, Cai X, Zhong H, Feng Q, Sunagawa S, et al An integrated catalog of reference genes in the human gut microbiome. Nat Biotechnol. 2014;32(8):834–41. Epub 2014/07/07. 10.1038/nbt.2942 . 24997786

[42] Treangen TJ, Koren S, Sommer DD, Liu B, Astrovskaya I, Ondov B, et al MetAMOS: a modular and open source metagenomic assembly and analysis pipeline. Genome Biol. 2013;14(1):R2 Epub 2013/01/17. 10.1186/gb-2013-14-1-r2 23320958 PMC4053804

[43] Bokulich NA, Rideout JR, Mercurio WG, Shiffer A, Wolfe B, Maurice CF, et al mockrobiota: a Public Resource for Microbiome Bioinformatics Benchmarking. mSystems. 2016;1(5). Epub 2016/11/09. 10.1128/mSystems.00062-16 27822553 PMC5080401

[44] Lim MY, Song EJ, Kim SH, Lee J, Nam YD. Comparison of DNA extraction methods for human gut microbial community profiling. Syst Appl Microbiol. 2018;41(2):151–7. Epub 2018/01/07. 10.1016/j.syapm.2017.11.008 . 29305057

[45] Wen Y, Xiao F, Wang C, Wang Z. The impact of different methods of DNA extraction on microbial community measures of BALF samples based on metagenomic data. Am J Transl Res. 2016;8(3):1412–25. Epub 2016/05/18. 27186268 PMC4858570

[46] Mao X, Cai T, Olyarchuk JG, Wei L. Automated genome annotation and pathway identification using the KEGG Orthology (KO) as a controlled vocabulary. Bioinformatics. 2005;21(19):3787–93. Epub 2005/04/09. 10.1093/bioinformatics/bti430 . 15817693

[47] Tatusov RL, Koonin EV, Lipman DJ. A genomic perspective on protein families. Science. 1997;278(5338):631–7. 10.1126/science.278.5338.631 PubMed PMID: WOS:A1997YC32300041. 9381173

[48] Finn RD, Coggill P, Eberhardt RY, Eddy SR, Mistry J, Mitchell AL, et al The Pfam protein families database: towards a more sustainable future. Nucleic Acids Research. 2016;44(D1):D279–D85. 10.1093/nar/gkv1344 PubMed PMID: WOS:000371261700038. 26673716 PMC4702930

